# B cell-intrinsic IRF-1 and conserved gammaherpesvirus protein kinase cooperate to promote murine gammaherpesvirus-driven germinal center response and splenic latent reservoir

**DOI:** 10.1128/jvi.01375-25

**Published:** 2025-11-20

**Authors:** Cade R. Rahlf, Christopher N. Jondle, Erika R. Johansen, Vera L. Tarakanova

**Affiliations:** 1 Department of Microbiology and Immunology, Medical College of Wisconsin 735651, Milwaukee, Wisconsin, USA; 2 Cancer Center, Medical College of Wisconsin 166013, Milwaukee, Wisconsin, USA; University of Virginia, Charlottesville, Virginia, USA

**Keywords:** gammaherpesvirus, germinal center B cells, chronic infection, gammaherpesvirus protein kinase, IRF-1

## Abstract

Gammaherpesviruses infect >90% of adults and are associated with B cell lymphomas. These viruses drive the expansion and differentiation of germinal center B cells to amplify latent viral reservoir and ensure life-long infection of memory B cells. Additionally, infected germinal center B cells seed viral lymphomagenesis. We previously demonstrated that global deficiency of Interferon Regulatory Factor-1 (IRF-1), a classically antiviral transcription factor, results in increased murine gammaherpesvirus-driven germinal center response and latent viral reservoir. In contrast, B cell-specific loss of IRF-1 expression attenuates murine gammaherpesvirus chronic infection and germinal center response. All gammaherpesviruses encode a conserved protein kinase. We previously showed that the murine gammaherpesvirus protein kinase antagonizes the antiviral activity of global IRF-1 expression to support the establishment of chronic infection. Intriguingly, in this study, we show cooperation between the gammaherpesvirus protein kinase and B cell-intrinsic IRF-1 expression that supported optimal establishment of splenic latent viral reservoir and murine gammaherpesvirus-driven germinal center response. Both viral protein kinase and B cell-intrinsic IRF-1 expression supported the survival and proliferation of germinal center B cells during chronic gammaherpesvirus infection. Elevated DNA damage response or Fas/FasL expression was not observed, failing to account for elevated apoptosis of IRF-1-deficient germinal center B cells. However, B cell-intrinsic IRF-1 deficiency led to decreased MHC-II expression by germinal center B cells during chronic gammaherpesvirus infection. In summary, the results of this study illustrate the critical role of the cell type in defining the functional outcome of the host-viral interaction.

**IMPORTANCE:**

Gammaherpesviruses are highly prevalent pathogens that uniquely target B cells for the establishment of life-long infection. This study demonstrates cooperation between a conserved gammaherpesvirus protein kinase and B cell-intrinsic IRF-1, a classical host antiviral factor. We show that this cooperation is critical to support proliferation and survival of germinal center B cells, a subset of B cells that is latently infected by gammaherpesviruses and is critical for both the establishment of chronic infection and viral lymphomagenesis.

## INTRODUCTION

Gammaherpesviruses are pervasive pathogens that uniquely infect and manipulate B cell biology to establish life-long infection. Human (Epstein-Barr virus, EBV) and murine (murine gammaherpesvirus 68, MHV68) gammaherpesviruses target naïve B cells in the secondary lymphoid organs to drive differentiation of both infected and bystander B cells through the germinal center response (1–3). Proliferation of infected germinal center B cells leads to rapid expansion of the viral latent reservoir; further differentiation into memory or plasma cells supports long-term latent infection or viral reactivation (4, 5). Importantly, gammaherpesvirus-driven germinal center response underlies viral pathogenesis as many EBV-positive B cell lymphomas bear evidence of germinal center differentiation (2). Given the species specificity of gammaherpesviruses, we and others have taken advantage of the tractable MHV68 experimental system to define host and viral factors that regulate gammaherpesvirus-driven germinal center response during natural infection of an intact host (6–19).

All gammaherpesviruses encode a conserved protein kinase. While a plethora of host and viral substrates of gammaherpesvirus protein kinases have been identified in cultured cells, the function of gammaherpesvirus protein kinases *in vivo* during chronic infection of an intact host is less understood. We previously demonstrated that expression and enzymatic function of a conserved gammaherpesvirus protein kinase encoded by MHV68 (*orf36*) facilitate the MHV68-driven germinal center response during chronic infection (8, 20). Correspondingly, the expression of orf36 encoded by human Kaposi’s sarcoma-associated herpesvirus (KSHV) was sufficient to drive B cell differentiation and eventual lymphomagenesis in transgenic mice in the absence of infection (21), highlighting an important role of the gammaherpesvirus protein kinases in manipulation of B cell differentiation.

In contrast to that observed for MHV68 orf36, global deficiency of Interferon Regulatory Factor 1 (IRF-1), a tumor suppressor and a founding member of the IRF family, led to an exaggerated germinal center response driven by chronic infection with MHV68, but not LCMV (10). Further, IRF-1 protein expression was selectively decreased in EBV-positive but not EBV-negative B cell lymphomas arising in transplant patients (10). Given the opposite germinal center phenotypes driven by loss of MHV68 orf36 or global IRF-1 expression, the interplay between MHV68 orf36 and IRF-1 was examined using a combination of viral and host genetic approaches. Interestingly, global IRF-1 deficiency rescued the attenuated germinal center response driven by the orf36 null MHV68 mutant (N36S) and partially rescued the N36S splenic reservoir (22), indicating an antagonistic relationship between MHV68 orf36 and global IRF-1 expression.

IRF-1 is ubiquitously expressed, with the expression further potentiated by several stimuli, including IFN signaling. While IRF-1 is expressed in B cells, its role in B cell biology remains far less explored as compared to its functions in the innate immune system. Given the observed antiviral phenotype of global IRF-1 expression during chronic MHV68 infection, we generated a mouse model of B cell-intrinsic IRF-1 deficiency by combining conditional *IRF-1* alleles with CD19 promoter-driven Cre recombinase knock-in allele (23). Surprisingly, and in contrast to that observed during global IRF-1 deficiency, B cell-intrinsic IRF-1 expression was proviral, as evidenced by decreased splenic latent reservoir and germinal center response driven by wild-type MHV68 infection in mice with IRF-1-deficient B cells (23).

The current study took advantage of a combination of host and viral genetics to define the mechanisms underlying the proviral role of B cell-intrinsic IRF-1 expression during chronic gammaherpesvirus infection and to determine the extent to which such mechanisms are modified by the expression of MHV68 protein kinase. Intriguingly, the proviral role of B cell-intrinsic IRF-1 expression in the establishment of MHV68 latent reservoir and MHV68-driven germinal center response was no longer evident in the absence of MHV68 orf36. A large proportion of MHV68 latent reservoir in the spleen is supported by infected germinal center B cells during the establishment of chronic infection. MHV68 orf36 and B cell-intrinsic IRF-1 expression independently promoted proliferation of germinal center B cells during chronic infection. In contrast, MHV68 orf36 and B cell-intrinsic IRF-1 played interdependent roles to attenuate the apoptosis of germinal center B cells. The observed increase in apoptosis of germinal center B cells was not accompanied by increased expression of Fas/FasL or increased activity of DNA damage response, a pro-apoptotic pathway activated in rapidly proliferating cells, such as germinal center B cells. However, B cell-intrinsic IRF-1 expression promoted germinal center B cell expression of MHC-II, a protein complex that mediates interaction between T follicular helper cells and germinal center B cells. Finally, and in contrast to that observed in the spleen, the previously reported antagonism between MHV68 orf36 and global IRF-1 expression was recapitulated in the peritoneal cells of mice with B cell-specific IRF-1 deficiency.

## RESULTS

### The interplay between conserved gammaherpesvirus protein kinase and B cell-intrinsic IRF-1 expression is modified by the anatomic site of infection

We previously demonstrated that the MHV68-encoded protein kinase orf36 antagonizes the antiviral function of global IRF-1 expression during chronic infection to promote the MHV68-driven germinal center response and the establishment of the latent reservoir (22). In contrast, we also showed that B cell-intrinsic IRF-1 expression is proviral during MHV68 infection as the establishment of chronic infection was attenuated in mice with B cell-specific IRF-1 deficiency following intranasal infection with 500 PFU of wild-type (WT) MHV68 (23). To define the relationship between MHV68 orf36 and B cell-intrinsic IRF-1 expression during chronic infection, parameters of chronic MHV68 infection were measured in *Cd19^Cre/wt^Irf1^fl/fl^* (Cre-positive) and *Cd19^wt/wt^Irf1^fl/fl^* (Cre-negative) mice 16 days after intranasal inoculation with 10,000 PFU of WT or orf36-deficient MHV68 mutant (N36S) (24). The 10,000 PFU dose was selected to enable meaningful comparisons of host parameters of infection as the N36S mutant fails to establish splenic latent reservoir at 16 days post-infection when inoculated at a lower 500 PFU dose used in our previous study (8, 23).

**Fig 1:**
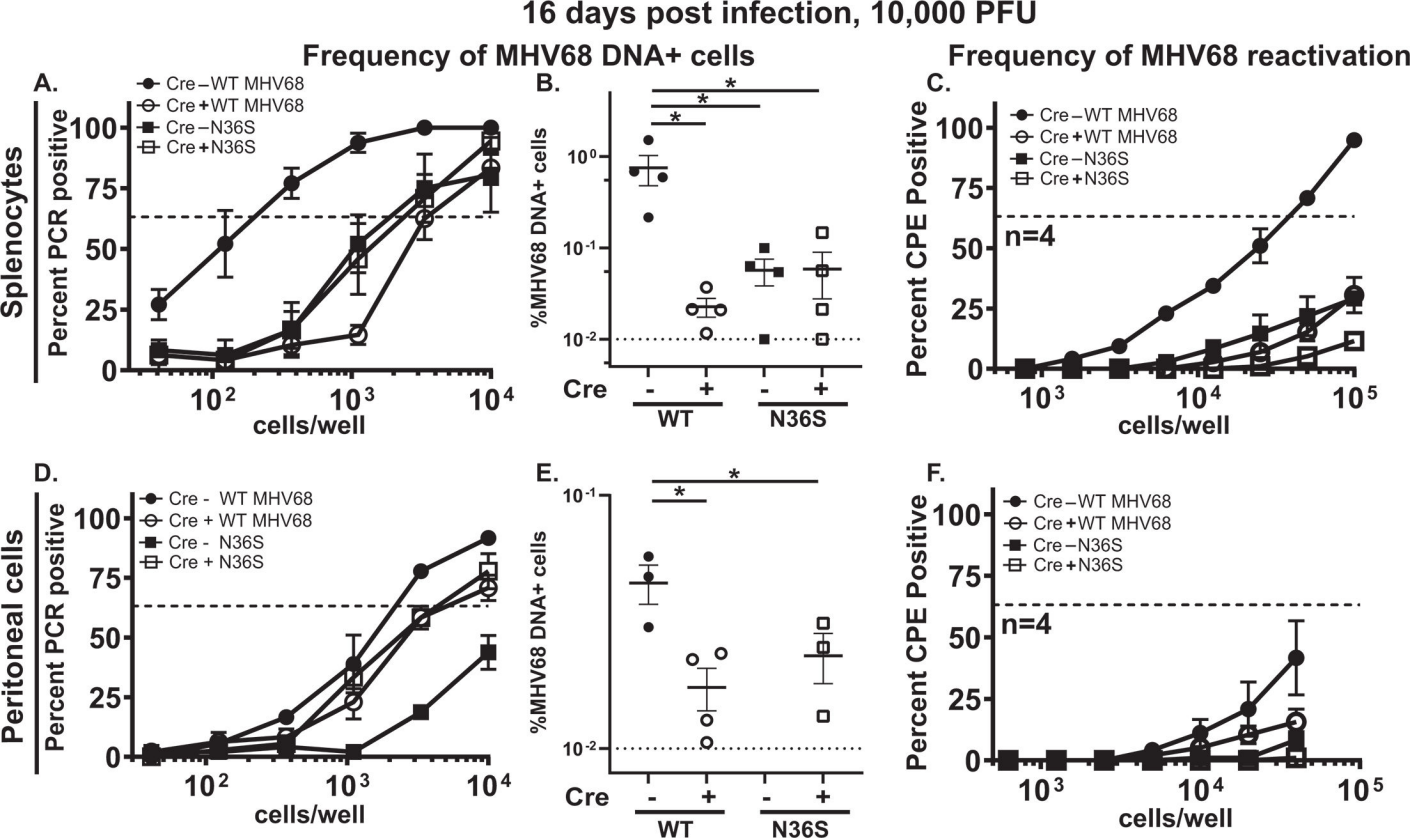
The interplay between conserved gammaherpesvirus protein kinase and B cell-intrinsic IRF-1 expression is modified by the anatomic site of infection. *Cd19^cre/wt^Irf1^fl/fl^* (Cre-positive) and *Cd19^wt/wt^Irf1^fl/fl^* (Cre-negative) mice were infected intranasally with 10,000 PFU of wild-type (WT) MHV68 or kinase-deficient (N36S) MHV68. At 16 days post-infection, single-cell suspensions of splenocytes (**A–C**) or peritoneal cells (**D–F**) were pooled from 3 to 4 mice/experimental group and subjected to limiting dilution assays to determine the frequencies of MHV68 DNA+ cells (**A, D**) and *ex vivo* reactivation (**C, F**). Data were pooled from three to four independent experiments. The intersection of the sigmoidal curve and the dotted line drawn at Y = 63.2% indicates the inverse frequency of positive events, as defined by the corresponding X coordinate. (**B, E**) The inverse frequency was converted to %MHV68 DNA+ cells, displayed for each independent study, and subjected to statistical analyses using ordinary one-way ANOVA followed by Tukey’s multiple comparison test; the dotted line represents the limit of quantitation. CPE, cytopathic effect. Mean and standard error of the mean are shown in this and subsequent figures. *P < 0.05.

As expected, the frequency and percent of MHV68 DNA-positive splenocytes were decreased in Cre-positive as compared to Cre-negative mice infected with WT MHV68 (Fig. 1A and B, compare groups with filled and open circles). The observed decrease (~25 fold) in the splenic latent reservoir of WT MHV68 following a 10,000 PFU inoculum was greater as compared to the previously published study, which used a lower inoculation dose of 500 PFU (~3 fold) (23), supporting the proviral role of B cell-intrinsic IRF-1 expression. Similar to that observed previously (8), the inability to express orf36 led to a significant decrease in the frequency and percent of the N36S-infected splenocytes of the control, Cre-negative mice (Fig. 1A and B, compare groups with filled circles and squares). Surprisingly, B cell-intrinsic IRF-1 deficiency had no effect on the latent reservoir of the N36S viral mutant, despite a profound attenuation of the WT MHV68 latent reservoir observed in Cre-positive mice. Thus, the proviral role of B cell-intrinsic IRF-1 in supporting the splenic latent reservoir was no longer evident under conditions when MHV68 orf36 could not be expressed. Likewise, the proviral role of orf36 required B cell-intrinsic IRF-1 expression, suggesting cooperation between the viral and host protein in the establishment of splenic latent reservoir.

Unlike germinal center B cells that host the majority of latent MHV68 reservoir in the spleen, plasma cells primarily support MHV68 reactivation from splenocytes of immunocompetent mice (5). Consistent with a decreased latent reservoir (Fig. 1A and B), reactivation of WT MHV68 was attenuated in Cre-positive as compared to Cre-negative splenocytes (Fig. 1C). As expected (8), reactivation of the N36S mutant was attenuated as compared to WT MHV68 in control Cre-negative splenocytes (Fig. 1C). Thus, MHV68 orf36 and B cell-intrinsic IRF-1 expression were both required to support MHV68 reactivation from splenocytes.

MHV68 establishes a latent reservoir in secondary lymphoid organs, such as the spleen, and in the body cavities, such as the peritoneal cavity. MHV68 infection in the spleen is intimately tied to the differentiation of splenic B cells, which represent the B-2 lineage. B-2 B cells undergo development in the bone marrow with subsequent MHV68-driven differentiation through a T cell-dependent germinal center response, which allows for exponential increase in the splenic latent reservoir (11, 25). In contrast, the majority of latent MHV68 in the peritoneal cavity is found in B-1 B cells, a distinct primordial B cell lineage in mice and humans (26). B-1 B cells develop in the embryonic yolk sac, self-renew, spontaneously produce self- and phospholipid-reactive antibodies, and primarily reside in body cavities, with limited circulation (reviewed in 27). When the MHV68 latent reservoir was examined in the peritoneal cavity, the frequency and percent of MHV68 DNA-positive peritoneal cells were decreased 2.7-fold in Cre-positive compared to Cre-negative mice infected with WT MHV68 (Fig. 1D and E, compare groups with open and filled circles). As expected, the lowest frequency of peritoneal cell infection was observed in Cre-negative mice infected with the N36S MHV68 mutant; the frequency was below the level of quantitation (Fig. 1D and E) (8, 22). In contrast to the splenic latent reservoir, loss of B cell-intrinsic IRF-1 expression resulted in a partial rescue of the peritoneal latent reservoir of the N36S viral mutant (Fig. 1D and E, compare groups with open and closed squares). Thus, similar to that observed under conditions of global IRF-1 deficiency (22), B cell-intrinsic IRF-1 deficiency partially rescued the attenuated peritoneal latent reservoir of the N36S mutant.

The low frequency of *ex vivo* WT MHV68 reactivation from peritoneal cells trended toward further decrease in Cre-positive as compared to Cre-negative mice (Fig. 1F), consistent with the decreased peritoneal latent reservoir. Interestingly, despite partial rescue of the peritoneal latent reservoir of the N36S viral mutant in the Cre-positive mice, *ex vivo* reactivation of the N36S mutant remained very low to undetectable regardless of the Cre genotype (Fig. 1F). No persistent viral replication was observed in the spleen or peritoneal cells in any of the control or experimental groups (data not shown).

In summary, B cell-intrinsic IRF-1 expression supported the establishment of the splenic latent reservoir; however, this proviral mechanism was dependent on the expression of the conserved MHV68 protein kinase. Similarly, the proviral role of MHV68 orf36 during the establishment of the splenic latent reservoir was dependent on the expression of B cell-intrinsic IRF-1, highlighting a cooperative relationship in B-2 B cells. In contrast, B cell-intrinsic IRF-1 expression was partially responsible for the attenuated peritoneal latent reservoir of the N36S MHV68 mutant, suggesting an antagonistic relationship between IRF-1 and MHV68 orf36 in B-1 B cells.

### B cell-intrinsic IRF-1 expression supports the orf36-driven expansion of the germinal center response and generation of class-switched antiviral and self-reactive antibodies

**Fig 2:**
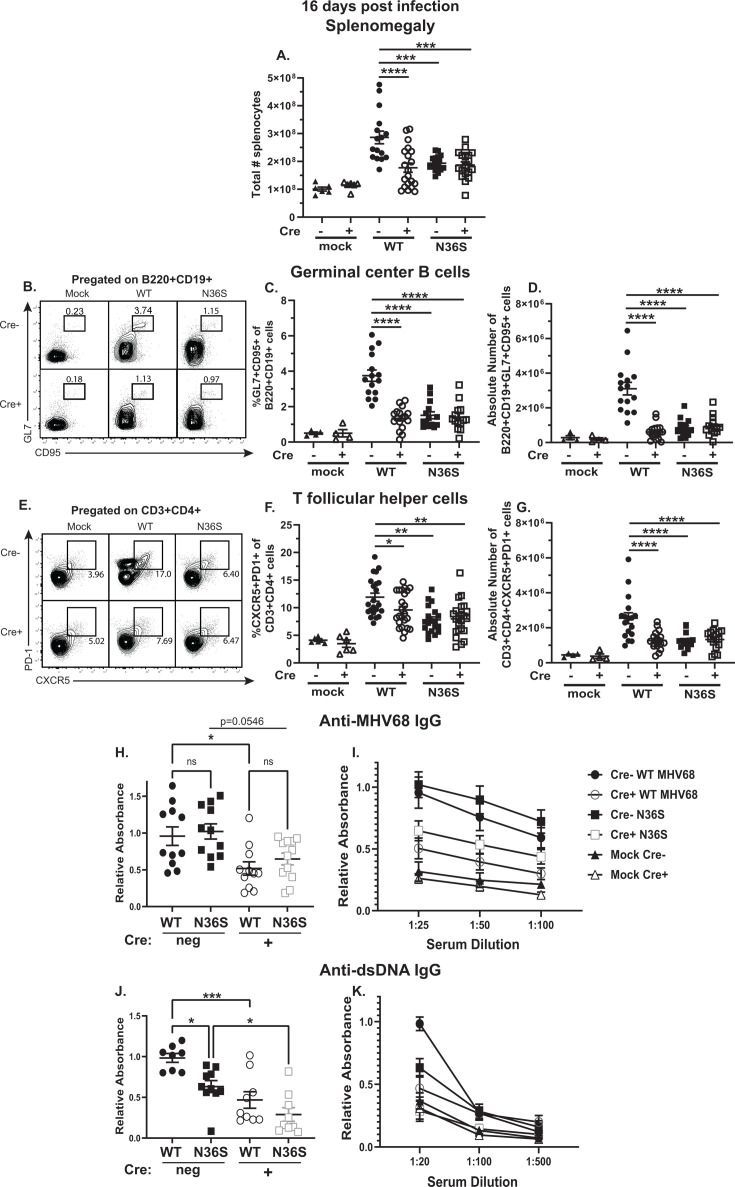
B cell-intrinsic IRF-1 expression supports the orf36-driven expansion of the germinal center response and generation of class-switched antiviral and self-reactive antibodies. *Cd19^cre/wt^Irf1^fl/fl^* (Cre-positive) and *Cd19^wt/wt^Irf1^fl/fl^* (Cre-negative) mice were infected as in Fig. 1 and analyzed at 16 days post-infection. (**A**) Absolute numbers of splenocytes. (**B**) Representative flow plot of germinal center B cells defined as B220^+^ CD19^+^ GL7^+^ CD95^+^ splenocytes. (**C, D**) Frequency and absolute numbers of germinal center B cells. (**E**) Representative flow plot of T follicular helper cells defined as CD3^+^ CD4^+^ PD-1^+^ CXCR5^+^ splenocytes. (**F, G**) Frequency and absolute numbers of T follicular helper cells. Each symbol represents an individual animal; data were pooled from four to five independent studies. (**H–K**) Sera were subjected to ELISA to determine titers of anti-MHV68 IgG (**H, I**) or anti-double-stranded DNA (dsDNA) IgG (**J, K**). (**H, J**) Relative absorbance of the least dilute serum (1:25 and 1:20 dilution, respectively); each symbol represents an individual animal. (**I, K**) Pooled relative absorbance data across all serum dilutions; data were pooled from three to four mice/group for the mock-infected animals. *P < 0.05; **P < 0.001; ***P < 0.0001; ****P < 0.00001.

Germinal center B cells host most of the MHV68 latent reservoirs at 16 days post-infection (25). The MHV68-driven expansion of germinal center B cells is dependent on CD4 T follicular helper cells (11, 28). Having observed the requirement of MHV68 orf36 for the proviral effects of B cell-intrinsic IRF-1 in the establishment of the splenic latent reservoir, the germinal center response was examined next. Splenomegaly, as defined by the absolute number of nucleated cells per spleen, was decreased in WT MHV68-infected Cre-positive as compared to Cre-negative mice (Fig. 2A). In contrast, B cell-specific IRF-1 deficiency did not result in a further decrease of an already attenuated splenomegaly observed in the N36S MHV68-infected mice (Fig. 2A). Consistent with decreased splenomegaly, the frequency and absolute number of germinal center B cells (Fig. 2B through D) and T follicular helper cells (Fig. 2E through G) were decreased in WT MHV68-infected Cre-positive compared to Cre-negative mice. Similarly, lack of MHV68 orf36 expression attenuated the germinal center response in control Cre-negative mice. However, B cell-intrinsic IRF-1 deficiency had no effect on the attenuated germinal center response driven by the N36S MHV68 mutant (Fig. 2B through G). Thus, like that observed for splenic latent reservoir, MHV68 orf36 and B cell-intrinsic IRF-1 expression served interdependent roles in supporting the MHV68-driven germinal center response.

In addition to physiological B cell differentiation that results in the generation of gammaherpesvirus-specific class-switched antibodies, EBV and MHV68 uniquely promote robust differentiation of B cells that produce class-switched antibodies against self- or foreign-species antigen. This nonphysiological B cell differentiation is thought to be proviral as EBV and MHV68 preferentially establish latency in B cells that do not encode a gammaherpesvirus-specific B cell receptor (29, 30). Having observed decreased germinal center response, humoral parameters of MHV68-driven B cell differentiation were defined next. As previously shown, MHV68 orf36 expression did not affect the generation of class-switched anti-MHV68 antibodies (Fig. 2H and I) (8). As expected (23), B cell-specific IRF-1 deficiency resulted in decreased anti-MHV68 IgG titers following WT MHV68 infection. Interestingly, the anti-MHV68 IgG titers trended toward decreased in Cre-positive as compared to Cre-negative mice infected with the N36S mutant (Fig. 2H and I), although this difference did not reach statistical significance (*P* = 0.0546), suggesting that B cell-intrinsic IRF-1 expression but not MHV68 orf36 supports anti-MHV68 IgG response.

We showed that, despite having no effect on the generation of anti-MHV68 antibody, MHV68 orf36 supports differentiation of self-reactive B cells (8). The same phenotype was observed in the current study, as reflected by the decreased anti-double-stranded DNA (dsDNA) IgG titers in N36S-infected Cre-negative mice (Fig. 2J and K). Further decreases in anti-ds DNA IgG were observed in Cre-positive mice infected with either WT or N36S MHV68 (Fig. 2J and K). Thus, both MHV68 orf36 and B cell-intrinsic IRF-1 expression supported MHV68-driven differentiation of self-reactive B cells.

### MHV68 orf36 and B cell-intrinsic IRF-1 expression independently support the MHV68-driven proliferation of splenic and germinal center B cells

MHV68 infection stimulates activation and proliferation of splenic B cells, including germinal center B cells, with the peak splenic B cell numbers observed at 14–16 days post-infection (29). Germinal center B cells undergo robust proliferation in the dark zone, with a 4–6 hour cell cycle (31), along with undergoing somatic hypermutation and isotype class-switching. Subsequently, germinal center B cells move to the light zone where they present antigens in the context of MHC-II to CD4 T follicular helper cells to receive survival and proliferative signals. The iterative dark-light zone cycles, if successful, result in further differentiation of germinal center B cells into memory B cells or antibody-secreting plasma cells. Failure to receive T cell help leads to apoptosis of germinal center B cells.

**Fig 3:**
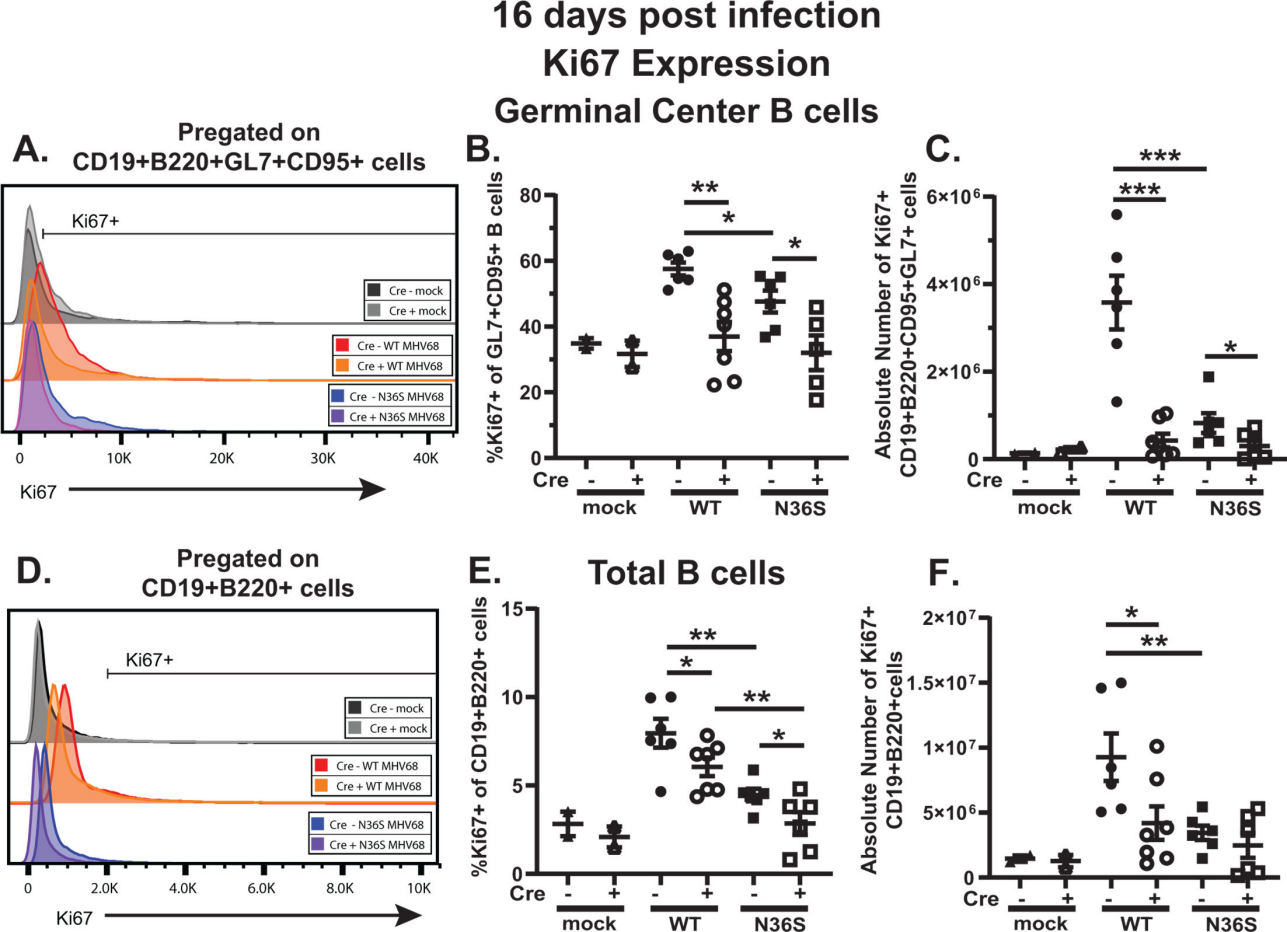
MHV68 orf36 and B cell-intrinsic IRF-1 expression independently support the MHV68-driven proliferation of splenic and germinal center B cells. *Cd19^cre/wt^Irf1^fl/fl^* (Cre-positive) and *Cd19^wt/wt^Irf1^fl/fl^* (Cre-negative) mice were infected as in Fig. 1 and splenocytes analyzed at 16 days post-infection using flow cytometry. (**A**) Representative histogram of proliferating germinal center B cells defined as Ki67^+^ CD19^+^ B220^+^ GL7^+^ CD95^+^ splenocytes. (**B, C**) Proportion and absolute number of proliferating germinal center B cells. (**D**) Representative histogram of proliferating splenic B cells defined as Ki67^+^ CD19^+^ B220^+^ splenocytes. (**E, F**) Proportion and absolute number of proliferating splenic B cells. Each symbol represents an individual animal; data are pooled from two to three independent experiments. *P < 0.05; **P < 0.001; ***P < 0.0001.

To determine the extent to which MHV68 orf36 and B cell-intrinsic IRF-1 expression affect B cell proliferation, Ki67 expression was assessed. As expected, significantly more germinal center B cells expressed Ki67 and at a higher level, as compared to total splenic B cells (compare Fig. 3A and D, please note the difference in the X-axis scale as the same gating strategy for Ki-67 is used in both panels). Specifically, approximately 60% of germinal center B cells in the spleens of WT MHV68-infected Cre-negative mice expressed Ki67 at 16 days post-infection, consistent with the increased MHV68-driven germinal center response (Fig. 3A through C). The proportion and number of Ki67+ germinal center B cells were decreased in Cre-negative mice infected with the N36S viral mutant, indicating that MHV68 orf36 expression facilitates proliferation of germinal center B cells (Fig. 3A through C). Surprisingly, and in contrast to the well-established antiproliferative role of IRF-1 in cancer (32), B cell-specific IRF-1 deficiency resulted in decreased proportion of Ki-67+ germinal center B cells. This was observed in both WT MHV68- and N36S mutant-infected Cre-positive mice (Fig. 3A through C). Similar phenotypes were observed when the overall splenic B cell population was examined (Fig. 3D through F). Thus, MHV68 orf36 and B cell-intrinsic IRF-1 expression independently promoted MHV68-driven splenic B cell proliferation, including that of germinal center B cells.

### MHV68 orf36 and B cell-intrinsic IRF-1 function within the same pathway to attenuate apoptosis of germinal center B cells

**Fig 4:**
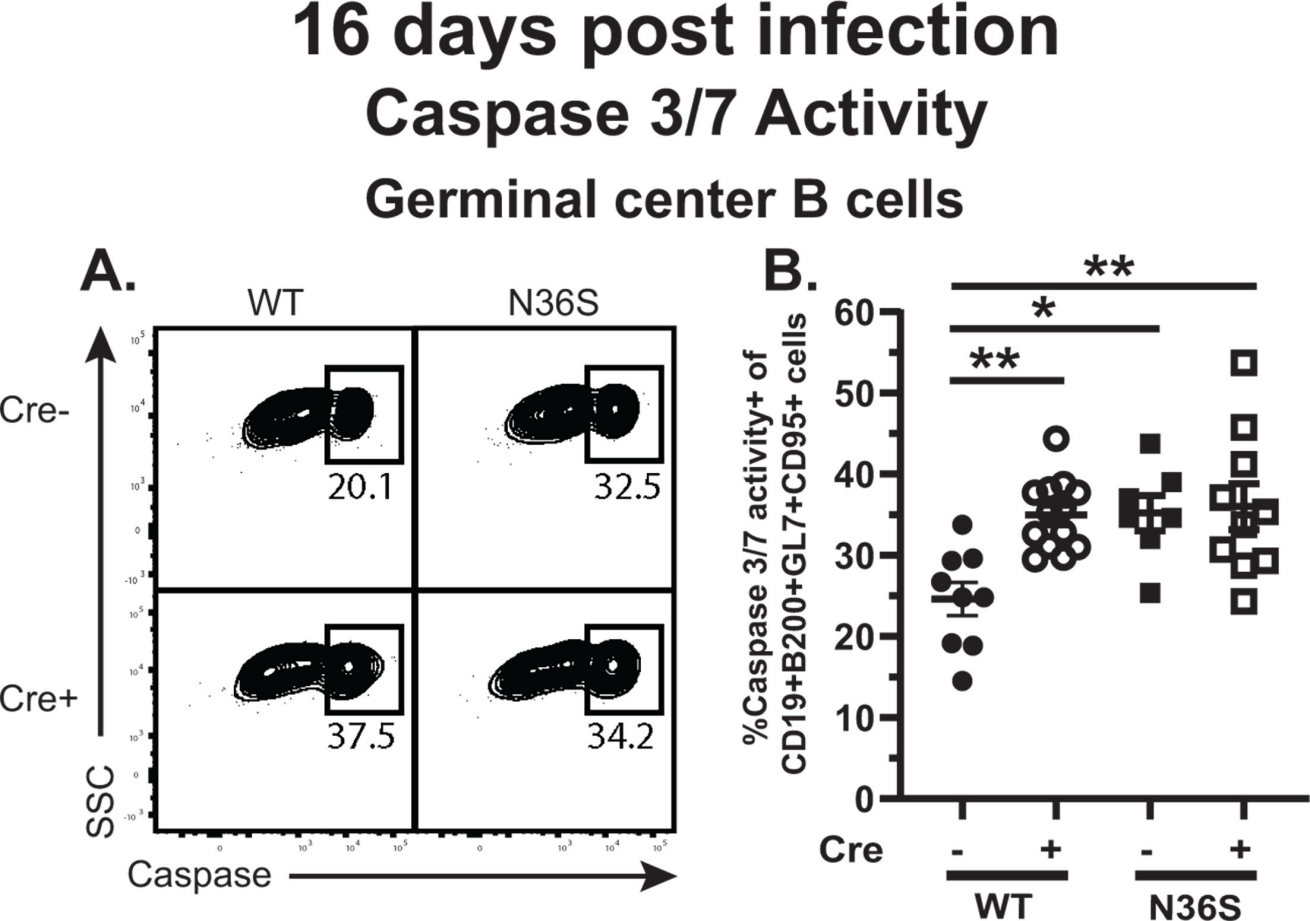
MHV68 orf36 and B cell-intrinsic IRF-1 function within the same pathway to attenuate the apoptosis of germinal center B cells. *Cd19^cre/wt^Irf1^fl/fl^* (Cre-positive) and *Cd19^wt/wt^Irf1^fl/fl^* (Cre-negative) mice were infected as in Fig. 1 and splenocytes analyzed at 16 days post-infection by flow cytometry. (**A**) Representative flow plot of caspase 3/7 activity in germinal center B cells defined as B220^+^ CD19^+^ GL7^+^ CD95^+^ splenocytes. (**B**) Proportion of germinal center B cells with active caspase 3/7. Each symbol represents an individual animal; data were pooled from three to four independent experiments. Comparisons were done using ordinary one-way ANOVA followed by Tukey’s multiple comparison test. *P < 0.05; **P < 0.001.

In addition to robust proliferation, a significant proportion of germinal center B cells undergoes apoptosis, via a combination of intrinsic apoptotic pathways, due to genomic instability and/or the paucity of T cell-mediated survival signals, and extrinsic apoptotic stimuli, such as ligation of Fas, expressed by B cells, by FasL, expressed by T cells. To quantify germinal center B cell apoptosis, the combined enzymatic activity of caspase 3 and 7 was assessed by flow cytometry. Loss of MHV68 orf36 or B cell-intrinsic IRF-1 expression resulted in increased proportion of germinal center B cells with active caspases (Fig. 4A and B). However, the proportion of germinal center B cells with active caspases remained similarly elevated in mice infected with the N36S MHV68 mutant, regardless of the IRF-1 expression by B cells. Thus, MHV68 orf36 and B cell-intrinsic IRF-1 functioned in the same pathway(s) to promote survival of germinal center B cells during chronic infection.

We previously showed that in contrast to decreased germinal center response observed in MHV68-infected mice with B cell-intrinsic IRF-1 deficiency, germinal center B cells were not decreased during chronic LCMV infection (23). To assess whether B cell-intrinsic IRF-1 expression selectively attenuated germinal center B cell apoptosis during MHV68 infection, Cre-negative and Cre-positive mice were immunized with sheep red blood cells (SRBC) that stimulate robust germinal center responses (33). SRBC immunization induced a similar magnitude of germinal center B cell population as that observed at 16 days post-MHV68 infection (compare Fig. S1A; Fig. 2B through D), with the proportion and number of germinal center B cells similar in Cre-negative and Cre-positive SRBC-immunized mice (Fig. S1A). In contrast to that observed in WT MHV68-infected control spleens, where ~ 25% of germinal center B cells expressed active caspases 3/7 (Fig. 4), only ~7% of germinal center B cells expressed active caspases in Cre-negative SRBC-immunized animals (Fig. S1B). Importantly, caspase 3/7 activity in germinal center B cells was not increased in SRBC-immunized Cre-positive mice (Fig. S1B). Thus, B cell-intrinsic IRF-1 expression selectively attenuated caspase 3/7 activity in germinal center B cells during chronic MHV68 infection.

### Increased apoptosis of germinal center B cells in the absence of MHV68 orf36 or B cell-intrinsic IRF-1 expression is not accompanied by increased DNA damage response

Having observed increased activity of apoptotic caspases in the absence of MHV68 orf36 or B cell-intrinsic IRF-1 expression, we sought to identify the mechanism underlying increased apoptosis. Germinal center B cells are highly susceptible to both intrinsic and extrinsic apoptotic stimuli. For the former, increased proliferation is inherently associated with DNA damage, including double-stranded DNA breaks, the most catastrophic DNA lesions that activate DNA damage response. Double-stranded DNA breaks are marked by phosphorylation of serine 139 of histone variant H2AX (termed as γH2AX), with phosphorylation conferred by several cellular kinases, including ataxia-telangiectasia mutated (ATM) kinase. In addition to allowing recruitment of DNA repair machinery, signaling initiated downstream of γH2AX leads to p53-dependent cell cycle arrest and, eventually, apoptosis. Classically, IRF-1 cooperates with both ATM and p53 to support cell cycle arrest (34, 35). Interestingly, MHV68 orf36 induces γH2AX directly and indirectly, the latter via activation of ATM, to support MHV68 lytic replication and establishment of chronic infection in a cell type-dependent manner (26, 36–39).

**Fig 5:**
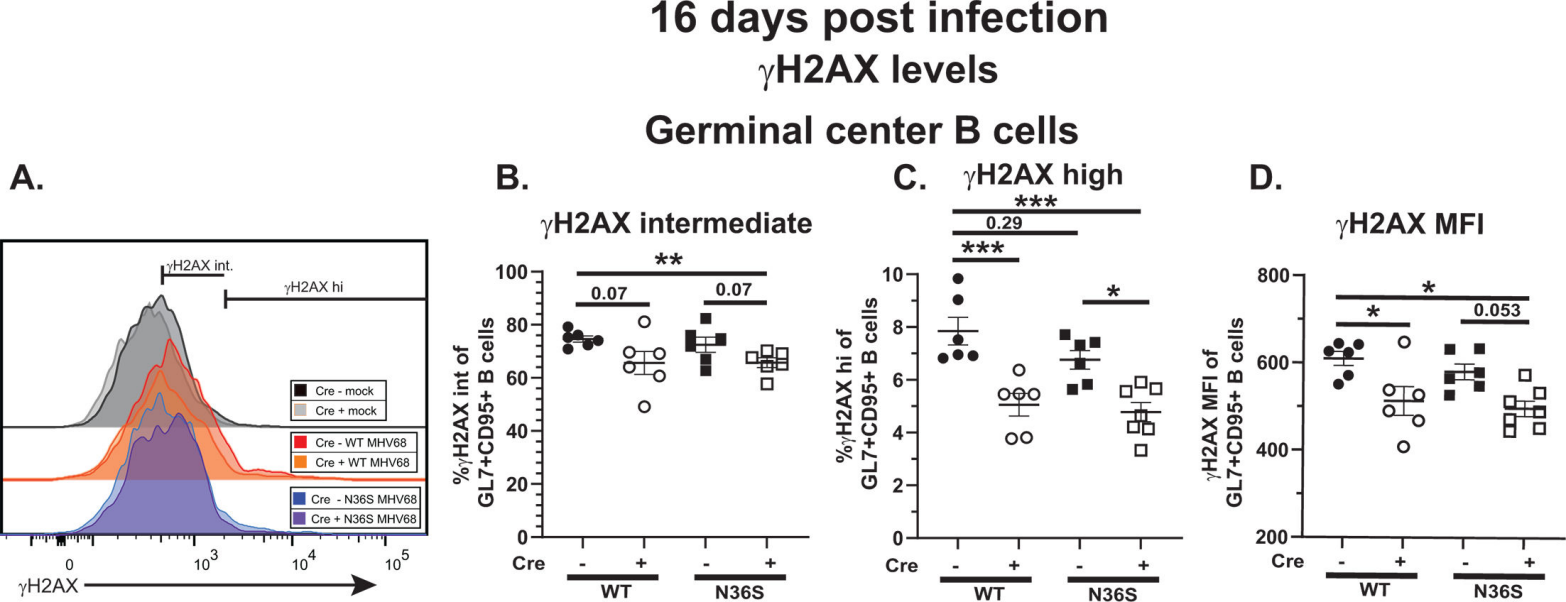
DNA damage response in germinal center B cells during chronic MHV68 infection in the absence of MHV68 orf36 or B cell-intrinsic IRF-1 expression. *Cd19^cre/wt^Irf1^fl/fl^* (Cre-positive) and *Cd19^wt/wt^Irf1^fl/fl^* (Cre-negative) mice were infected as in Fig. 1 and splenocytes analyzed at 16 days post-infection by flow cytometry. (**A**) Representative flow plot of γH2AX levels in germinal center B cells defined as in Fig. 2A. (**B, C**) Proportion of germinal center B cells with intermediate (**B**) or high (**C**) γH2AX levels. (**D**) Median fluorescence intensity of γH2AX in the overall germinal center B cell population. Each symbol represents an individual animal; data were pooled from two to three independent experiments. *P < 0.05; **P < 0.001, ***P < 0.0001.

To determine the extent to which DNA damage response activity in germinal center B cells is modulated during chronic MHV68 infection, we optimized γH2AX detection by flow cytometry. Optimization was performed using UV-irradiated or untreated splenocytes from naïve mice (Fig. S2). γH2AX levels in UV-irradiated B cells were used to establish the flow cytometry gating strategy to identify B cell populations with intermediate (γH2AX int) and high (γH2AX hi) γH2AX levels (Fig. S2). This gating strategy was applied to quantify γH2AX levels in germinal center B cells analyzed directly *ex vivo* at 16 days post-infection (Fig. 5).

Approximately 70% of all germinal center B cells in Cre-negative mice infected with WT MHV68 displayed intermediate γH2AX levels (Fig. 5B), consistent with ~60% of proliferating, Ki67 +germinal center B cells observed under the same conditions (Fig. 3B). Lack of MHV68 orf36 expression did not affect the frequency and number of germinal center B cells with intermediate γH2AX levels (Fig. 5A and B). Surprisingly, and in contrast to the classical role of IRF-1 in supporting DNA repair (40), the frequency of germinal center B cells with intermediate γH2AX levels was decreased in mice with B cell-specific IRF-1 deficiency regardless of the MHV68 genotype (Fig. 5B). Significantly fewer (~5%–10%) germinal center B cells displayed high γH2AX levels, with decreased proportion of γH2AX high germinal center B cells observed in Cre-positive mice (Fig. 5C). Finally, the median fluorescence intensity of the γH2AX signal in the overall germinal center B cell population was also reduced in mice with B cell-specific IRF-1 deficiency. Thus, increased apoptosis of germinal center B cells in the absence of MHV68 orf36 or B cell-intrinsic IRF-1 expression was not accompanied by the increase in the DNA damage response.

### MHV68 orf36 or B cell-intrinsic IRF-1 expression does not alter Fas or Fas ligand (FasL) expression on germinal center B cells during chronic infection

**Fig 6:**
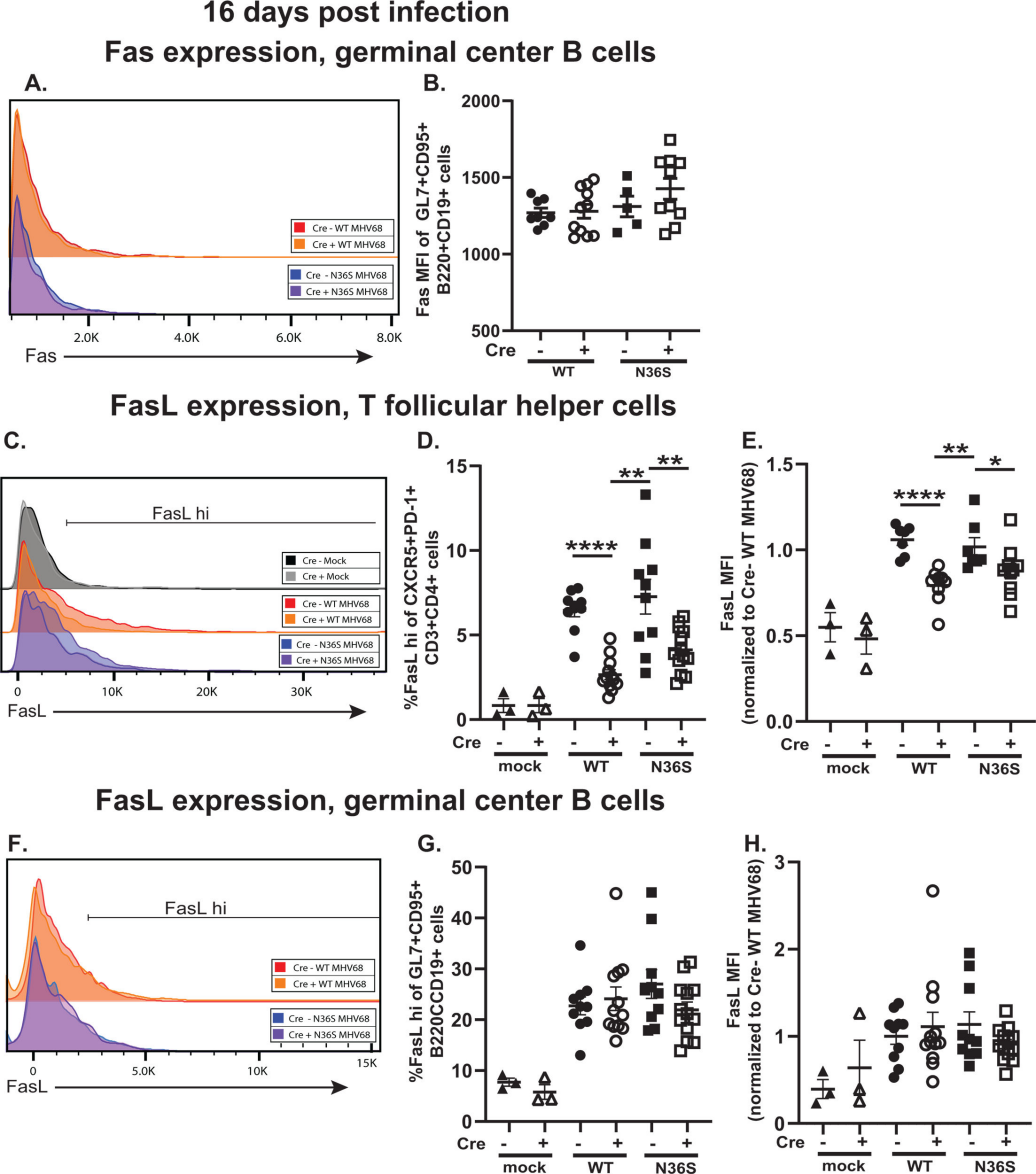
MHV68 orf36 or B cell-intrinsic IRF-1 expression does not alter Fas or Fas ligand (FasL) expression on germinal center B cells during chronic infection. *Cd19^cre/wt^Irf1^fl/fl^* (Cre+) and *Cd19^wt/wt^Irf1^fl/fl^* (Cre-) mice were infected as in Fig. 1 and splenocytes analyzed by flow cytometry at 16 days post-infection. (**A**) Representative histogram of Fas levels expressed by germinal center B cells defined as in Fig. 2A. (**B**) Median fluorescence intensity (MFI) of Fas (CD95) protein levels expressed by germinal center B cells. (**C**) Representative histogram of FasL protein levels expressed by T follicular helper cells defined as in Fig. 2D. (**D**) Proportion of T follicular helper cells with high FasL expression. (**E**) Relative MFI of FasL protein levels expressed by T follicular helper cells. FasL MFIs were normalized to the WT MHV68-infected Cre-negative group within each study (**E, H**). (**F**) Representative histogram of FasL protein levels expressed by germinal center B cells; the gating strategy was adjusted as compared to panel C to accommodate the overall lower expression of FasL by germinal center B cells as compared to T follicular helper cells. (**G**) Proportion of germinal center B cells with high FasL expression. (**H**) Relative MFI of FasL protein levels expressed by germinal center B cells. Each symbol represents an individual animal; data were pooled from two to three independent experiments. *P < 0.05; **P < 0.001; ***P < 0.0001; ****P < 0.00001.

In the absence of increase in the DNA damage response in orf36- or IRF-1-deficient conditions, extrinsic apoptotic stimuli were examined next. FasL is expressed by most activated and differentiated CD4 T cells, including germinal center-localized CD4 T follicular helper cells, with corresponding cytotoxic activity (41). Germinal center B cells are exquisitely susceptible to Fas-mediated apoptosis; in fact, high expression of CD95 (Fas) is a defining cell surface marker for immunophenotyping of germinal center B cells (Fig. 2B). However, neither MHV68 orf36 nor B cell-intrinsic IRF-1 expression altered Fas protein levels on the cell surface of germinal center B cells at 16 days post-infection (Fig. 6A and B).

When FasL expression was examined, both the proportion of T follicular helper cells with high FasL expression and the per cell FasL protein expression levels were decreased in infected Cre-positive mice, regardless of the infecting virus (Fig. 6C through E). This was surprising as only B cells are genetically targeted in this mouse model. However, the observed decreased FasL expression by T follicular helper cells in the absence of B cell-intrinsic IRF-1 expression did not explain increased apoptosis of germinal center B cells under the same conditions (Fig. 4). Thus, expression of FasL by germinal center B cells was examined next. In contrast to T cells, expression of FasL by B cells, including germinal center B cells, is poorly defined, including functionally. Interestingly, FasL expression by germinal center B cells increased following MHV68 infection, albeit to a lesser extent as compared to T follicular helper cells (Fig. 6C vs. F, note the different X-axis scale and gating strategy). However, neither MHV68 orf36 nor B cell-intrinsic IRF-1 expression affected FasL cell surface protein levels or the proportion of FasL-expressing germinal center B cell population (Fig. 6F through H). Thus, neither B cell-intrinsic IRF-1 deficiency nor MHV68 orf36 expression affected Fas/FasL levels expressed by germinal center B cells.

### B cell intrinsic IRF-1 but not MHV68 orf36 supports MHCII expression on germinal center B cells during chronic MHV68 infection

**Fig 7:**
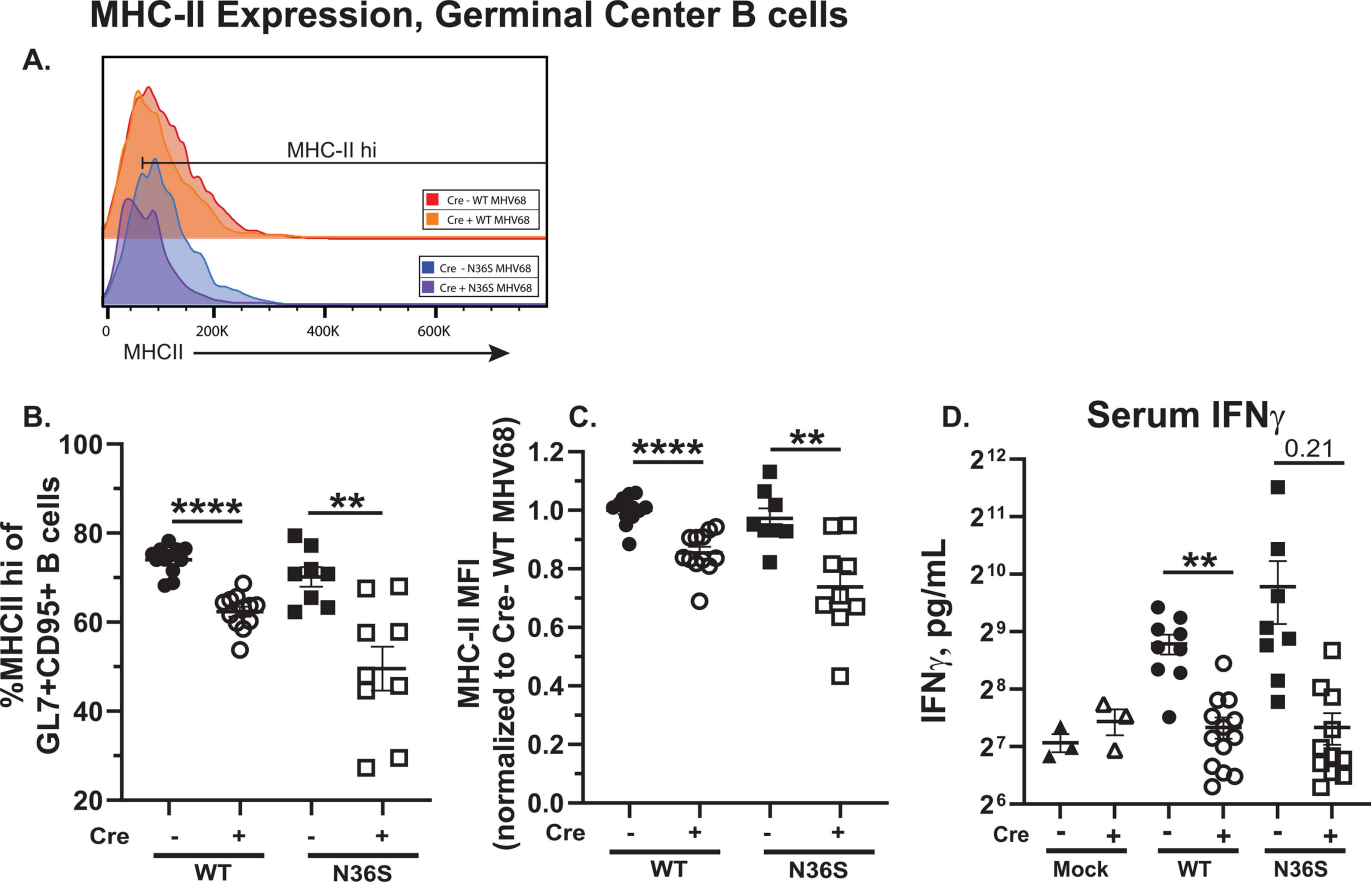
B cell intrinsic IRF-1, but not MHV68 orf36, supports MHCII expression by germinal center B cells during chronic MHV68 infection. *Cd19^cre/wt^Irf1^fl/fl^* (Cre-positive) and *Cd19^wt/wt^Irf1^fl/fl^* (Cre-negative) mice were infected as in Fig. 1 and analyzed at 16 days post-infection. (**A**) Representative histogram of MHC-II protein levels expressed by germinal center B cells defined as in Fig. 2A. (**B**) Frequency of germinal center B cells with high MHC-II expression. (**C**) MFI of MHC-II protein levels expressed by germinal center B cells. MHC-II MFIs were normalized to the WT MHV68-infected Cre-negative group within each study. (**D**) Serum IFNγ levels. Each symbol represents an individual animal; data were pooled from two to three independent experiments. **P < 0.001; ****P < 0.00001.

Under physiological conditions, MHCII-dependent antigen presentation to CD4 T follicular helper cells is critical for the survival and further differentiation of germinal center B cells (42). In the context of MHV68 infection, both B cell-intrinsic MHCII expression and T follicular helper cells are necessary to support MHV68-driven splenic B cell differentiation (11, 28, 43). Because IFNγ signaling significantly increases MHC-II expression via the IRF-1/CIITA axis (44), with peak serum IFNγ levels observed at 16 days post-MHV68 infection (45), the expression of MHC-II by germinal center B cells was measured next. Both per cell protein levels of MHC-II and proportion of germinal center B cells with high MHC-II expression were decreased in mice with B cell-specific IRF-1 deficiency (Fig. 7A through C). In contrast, lack of orf36 did not affect MHC-II expression by germinal center B cells (Fig. 7A through C). The differences in MHC-II expression observed in germinal center B cells were mirrored by the differences in the serum IFNγ levels, although the IRF-1-dependent difference in the N36S-infected mice did not reach statistical significance (Fig. 7D, *P* = 0.2086). Thus, B cell-intrinsic IRF-1 expression supported germinal center B cell MHC-II expression during chronic MHV68 infection, independent of MHV68 protein kinase.

## DISCUSSION

Gammaherpesviruses are ubiquitous, oncogenic pathogens that, unlike other viral families, specifically target and manipulate B cells to establish lifelong infection. Gammaherpesvirus-driven germinal center-based B cell differentiation supports the establishment of life-long infection in memory B cells and seeds viral lymphomagenesis. We previously demonstrated that conserved gammaherpesvirus protein kinase and global expression of host IRF-1 exert opposite and antagonistic effects on gammaherpesvirus-driven germinal center response and latent viral reservoir during chronic infection (8, 10, 22). Surprisingly, using a mouse model of B cell-intrinsic IRF-1 deficiency, we also showed that IRF-1 expression by B cells is proviral and supports the establishment of chronic gammaherpesvirus infection and germinal center response (23). Thus, the current study tested an intriguing hypothesis that B cell-intrinsic IRF-1 may be subverted by MHV68 using the conserved gammaherpesvirus protein kinase. Indeed, we found that the proviral functions of B cell-intrinsic IRF-1 in supporting germinal center response and MHV68 splenic latent reservoir required the expression of MHV68 orf36. While IRF-1 and orf36 expression independently supported proliferation of germinal center B cells during chronic infection, unexpectedly, IRF-1 attenuated apoptosis of germinal center B cells in an orf36-dependent manner. The cooperation between MHV68 orf36 and B cell-intrinsic IRF-1 in germinal center B cell survival was not explained by corresponding changes in the DNA damage response or Fas/FasL expression. Importantly, B cell-intrinsic IRF-1 expression, but not MHV68 orf36, supported optimal expression of MHC-II by germinal center B cells, a critical requirement to receive survival and proliferation stimuli from T follicular helper cells and promote MHV68-driven B-cell differentiation (11, 42). Overall, the outcomes of the current study demonstrate the important role of the host cell type in defining functional consequences of viral-host interactions.

We previously showed that neither global nor B cell-specific IRF-1 expression affected B cell differentiation during chronic infection with an unrelated virus (LCMV). Thus, the novel role(s) of IRF-1 and/or IRF-1 cooperation with MHV68-specific viral functions/proteins may be responsible for IRF-1-dependent host phenotypes that selectively manifested during MHV68 infection. The latter is likely as we show that several viral and host IRF-1-dependent phenotypes required the expression of MHV68 orf36 (as summarized in Table 1). Of these phenotypes, B cell-intrinsic IRF-1 expression promoted survival of germinal center B cells, but only when MHV68 orf36 expression was preserved. This was not observed in the context of SRBC immunization, where the proportion of apoptotic germinal center B cells trended toward decreased in mice with IRF-1-deficient B cells. The observed apoptosis phenotypes in immunized mice with IRF-1-deficient B cells are consistent with the classical role of IRF-1 in promoting p53-dependent apoptosis and cell cycle arrest. In contrast, the observed increase in apoptosis of IRF-1-deficient germinal center B cells during chronic MHV68 infection is opposite to the classical IRF-1 functions.

**TABLE 1:** Interplay of MHV68 orf36 and B cell-intrinsic IRF-1 expression in the observed viral and host phenotypes of chronic MHV68 infection

Viral or host parameter	Independent roles	Interdependent roles
Splenic latent reservoir (Fig. 1)		Proviral roles of MHV68 orf36 and B cell-intrinsic IRF-1 require the expression of both proteins
Peritoneal latent reservoir (Fig. 1)		Antagonism between IRF-1 and MHV68 orf36
MHV68-driven germinal center response (Fig. 2)		Support of MHV68-driven germinal center response by MHV68 orf36 and B cell-intrinsic IRF-1 requires the expression of both proteins
Anti-MHV68 IgG titers (Fig. 2)	Antiviral IgG titers are supported by B cell-intrinsic IRF-1, but not MHV68 orf36 expression	
Anti-double-stranded DNA IgG titers (Fig. 2)	Self-reactive IgG titers are supported by MHV68 orf36 or B cell-intrinsic IRF-1 expression.	
Germinal center B cell proliferation (Fig. 3)	Germinal center B cell proliferation is supported by MHV68 orf36 or B cell-intrinsic IRF-1 expression.	
Germinal center B cell apoptosis (caspase 3 activity, Fig. 4)		Attenuation of germinal center B cell apoptosis by MHV68 orf36 and B cell-intrinsic IRF-1 requires the expression of both proteins
Germinal center B cell DNA damage response (Fig. 5)	DNA damage response of germinal center B cells is attenuated in the absence of B cell-intrinsic IRF-1 expression	
MHC-II expression by germinal center B cells (Fig. 7)	MHC-II expression is supported by B cell-intrinsic IRF-1, but not MHV68 orf36 expression	

The current study probed potential IRF-1-dependent mechanisms that would support IRF-1-dependent germinal center B cell survival during MHV68 infection. Having ruled out DNA damage response and Fas/FasL system, decreased MHC-II expression by germinal center B cells was observed in infected mice with IRF-1-deficient B cells. Concurrently, there was a statistically significant or a trending decrease in serum IFNγ levels of infected Cre-positive mice (Fig. 7D). This decreased MHC-II expression offers one possible scenario by which the classical IFNγ/IRF-1/CIITA/MHC-II axis facilitates optimal interactions between germinal center B cells and T follicular helper cells, with the latter delivering survival and proliferation stimuli to B cells. Interestingly, SRBC immunization preferentially induces the type I IFN response *in vivo* (46), which, unlike type II IFN, plays a minimal role in stimulating MHC-II expression. Thus, it is conceivable that the lack of IRF-1-dependent germinal center phenotype following SRBC immunization (Fig. S1) is due to selective induction of type I IFN in immunized mice. Importantly, the regulation of MHC-II expression was not affected by the orf36 MHV68 genotype, failing to explain the observed dependence of IRF-1-mediated germinal center B cell survival on MHV68 orf36. Thus, future studies are needed to tease out the involved mechanisms.

IRF-1 can interact with multiple transcription factors, both ubiquitous and expressed in a cell type-dependent or inducible manner (47). Thus, IRF-1 target genes have to be defined in individual cell types and under physiologically relevant conditions. A single published study from the Lund group comprehensively defined IRF-1-dependent gene expression in transitional, follicular, and marginal zone B cell subsets of unmanipulated mice (48). Gene signature of IRF-1-deficient transitional B cells was indicative of an increased activity of NF-kB and type I IFN pathways (48). However, NF-kB is a critical host factor usurped by gammaherpesviruses, including MHV68, to establish latent infection (16, 49, 50). Similarly, we showed that B cell-intrinsic expression of STAT1, a transcription factor required for classical IFN antiviral responses, supports the establishment of latent MHV68 reservoir in the spleen (19). While it is possible that exaggerated NF-kB and IFN activity in IRF-1-deficient germinal center B cells is antiviral, this remains to be confirmed in the context of MHV68 infection, along with comprehensive analyses of IRF-1 target genes in germinal center B cells.

We have also, for the first time, defined the role of MHV68 orf36 in the biology of germinal center B cells during chronic infection. The current study demonstrates that MHV68 orf36 expression promotes the proliferation and survival of germinal center B cells during chronic infection, the former independent of and the latter in collaboration with IRF-1 expressed by B cells. Our observations suggest that orf36 is expressed and functions in germinal center B cells. This hypothesis is highly provocative as gammaherpesvirus protein kinases are classically defined as lytic cycle-associated proteins, in contrast to the tightly latent infection of germinal center B cells. Importantly, a recent publication from the Krug group profiled MHV68 gene expression by bulk RNA sequencing of sorted infected germinal center B cells and, indeed, detected orf36 transcript, albeit at low levels (51). In the future, it will be important to develop new tools to define the timing of MHV68 orf36 expression in specific germinal center B cell subsets in an intact animal. We previously showed that MHV68 orf36 antagonizes STAT1 antiviral function in myeloid cells to support MHV68 passage to splenic B cells (20). Thus, in a non-exclusive scenario, orf36 expression in specific infected myeloid cell types may promote the differentiation of T follicular helper cells and subsequent germinal center responses in a manner that subverts IRF-1-dependent processes.

The results of the current study underscore the complexity of the interactions between classically antiviral transcription factors and viral-encoded proteins that manipulate the physiological immune response. IRF-1 is evolutionarily conserved, representing an ancient component of the innate immune response. Likewise, gammaherpesviruses are ancient viruses, having evolved alongside the development of the modern immune response. As gammaherpesviruses, such as EBV, rely on establishing infection in host B cells, it is no surprise that these viruses are masters in manipulating and usurping the host immune response. Our study suggests that gammaherpesviruses have evolved a multifunctional conserved protein kinase to usurp the functions of the canonically antiviral transcription factor to promote the establishment of the viral latent reservoir in splenic B cells. In contrast, results of the current study confirm the antagonistic role of MHV68 orf36 and B cell-intrinsic IRF-1 in the context of peritoneal cavity infection. Thus, not only are viral-host interactions modified in a cell type-dependent manner, the B cell lineage (B-2 vs B-1) also plays a role. This is not an unprecedented finding as we showed that B cell-intrinsic STAT1 and IFNAR1 expression exert opposite effects on the establishment of MHV68 latent reservoir in splenic vs peritoneal B cells (19). IRF-1 expression is increased downstream of IFN receptors, and IRF-1 can complex with STAT1 to regulate gene expression (47). Thus, it is possible that the viral and host phenotypes driven by B cell-intrinsic IRF-1 deficiency in this study reflect the IRF-1-/STAT1-dependent changes in B cell-intrinsic gene expression, to be defined in the future.

## MATERIALS AND METHODS

### Animal studies

All mice were housed and bred in a specific-pathogen-free facility at MCW. *Cd19^cre/wt^IRF-1^loxP/loxP^* mice were previously described and validated (23). The presence of the conditional IRF-1 allele was detected using TGTTCTAGCAAGTTCTCAGAGG (forward) and TGGTACCCTGACTCACAACTG (reverse) primers. The presence of the CD19 Cre recombinase allele was detected using ACGTACTGACGGTGGAGAA (forward) and CAAAAATCCCTTCCAGGGCG (reverse) primers.

### Virus infection

Virus stock titers were determined by plaque assay on NIH 3T12 cells. Infections with the N36S MHV68 mutant were controlled by the parental virus retaining a single LoxP site (referred to as wild-type in the figures and text) (24). Mice between the ages of 8 and 10 weeks were intranasally inoculated (15 µL/mouse) with 10,000 PFU of virus diluted in sterile serum-free Dulbecco’s modified Eagle’s medium (Corning, Tewksbury, MA) or sterile carrier (mock) under light anesthesia.

### SRBC inoculation

Mice between the ages of 8 and 10 weeks were inoculated via intraperitoneal injection with 300 uL of fresh sheep red blood cells (Colorado Serum Company, Denver, CO). Splenocytes from individual mice were analyzed at 9 days post-immunization.

### Limiting dilution assays

The frequency of MHV68 DNA+ cells was determined as previously described (52). Briefly, splenocytes or peritoneal cells were pooled from each experimental group (3–5 mice/group), and six 3-fold dilutions were made on a background of NIH 3T12 cells. Dilutions were subjected to a nested PCR (12 replicates/dilution) using primers designed against the MHV68 genome (outer forward: 5′-GAGATCTGTACTCAGGCACCTGT-3′; outer reverse: 5′-GGATTTCTTGACAGCTCCCTGT-3′; inner forward: 5′-TGTCAGCTGTTGTTGCTCCT-3′; inner reverse: 5′-CTCCGTCAGGATAACAACGTCT-3′). To determine the frequency of *ex vivo* MHV68 reactivation, 2-fold serial dilutions of pooled splenocytes or peritoneal cells were plated onto a monolayer of C57BL6/J mouse embryonic fibroblasts (MEFs) at 24 replicates per dilution and incubated at 37°C. To control for preformed virus, 2-fold serial dilutions of mechanically disrupted cells were plated on MEFs. Viral reactivation, as indicated by cytopathic clearing of MEFs, was assessed on day 21 of culture. The frequency of MHV68 DNA+ cells or *ex vivo* reactivation is determined by Poisson distribution.

### Flow cytometry

Single-cell suspensions of splenocytes were prepared in fluorescence-activated cell sorter (FACS) buffer (phosphate-buffered saline, 2% fetal bovine serum); 2 × 10^6^ cells were treated with Fc block prior to extracellular staining with optimized antibody concentrations for 30 minutes on ice. For intracellular detection of γH2AX, cell permeabilization was performed using FOXP3 Fix/Perm Buffer Set (cat: 421403; BioLegend (San Diego, CA)) followed by 1 hour incubation with optimized antibody concentration at room temperature. Ki67 staining followed the BioLegend Ki-67 Flow Cytometry Staining Protocol, where cells were permeabilized by incubating at −20°C with 70% EtOH for 2 hours, followed by a 30 minute incubation with an optimized antibody concentration at room temperature. Data were acquired using Celesta flow cytometer (BD Biosciences, Franklin Lakes, NJ) and analyzed using FlowJo software (BD Biosciences, Franklin Lakes, NJ). The following list of antibodies used in this study were purchased from BioLegend (San Diego, CA): CD19-Bv421 (cat. 152415), B220-PE/Cy7 (cat. 103222), GL7-PerCP/Cy5.5 (cat. 144609), GL7-FITC (cat. 144605), CD95-PECF594 (cat. 562499), CD3-Bv421 (cat. 100531), CD4-FITC (cat. 100406), CXCR5-PECF594 (cat. 145522), PD-1-Bv605 (cat. 135220), MHCII-Bv605 (cat. 107639); Invitrogen (Carlsbad, CA): Ki67-PE (cat. 12-5698-82), FasL-APC (cat. 17-5911-82), Caspase-3/-7 Green Flow Cytometry Assay Kit (cat. C10427); or Cell Signaling Technology (Danvers, MA): yH2AX-Bv421 (cat. 9718).

### ELISA

ELISA was performed to measure serum IgM, IgG, MHV68-specific IgG, and anti-double-stranded DNA IgG, as previously described (22).

### Statistical analyses

Statistical analyses were performed using Student *t*-test when comparing two groups, and one-way ANOVA with Tukey’s *post hoc* test when comparing more than two groups (Prism, GraphPad Software, Inc.).

## Data Availability

All data associated with the study are available directly in the article.

## SUPPLEMENTAL MATERIAL

Figure S1 jvi.01375-25-s0001.pdf Sheep red blood cell immunization.

Figure S2 jvi.01375-25-s0002.pdf Gating strategy for DNA damage response marker.

ASM does not own the copyrights to Supplemental Material that may be linked to, or accessed through, an article. The authors have granted ASM a non-exclusive, world-wide license to publish the Supplemental Material files. Please contact the corresponding author directly for reuse.
