# Supplementary material for: B cell-intrinsic IRF-1 and conserved gammaherpesvirus protein kinase cooperate to promote murine gammaherpesvirus-driven germinal center response and splenic latent reservoir
Source: J Virol. 2025 Nov 20;99(12):e01375-25. doi: 10.1128/jvi.01375-25 (PMC12724244; doi:10.1128/jvi.01375-25)
Supplement: Figure S1 — Sheep red blood cell immunization. [file jvi.01375-25-s0001.pdf]

Supplemental Figure 1

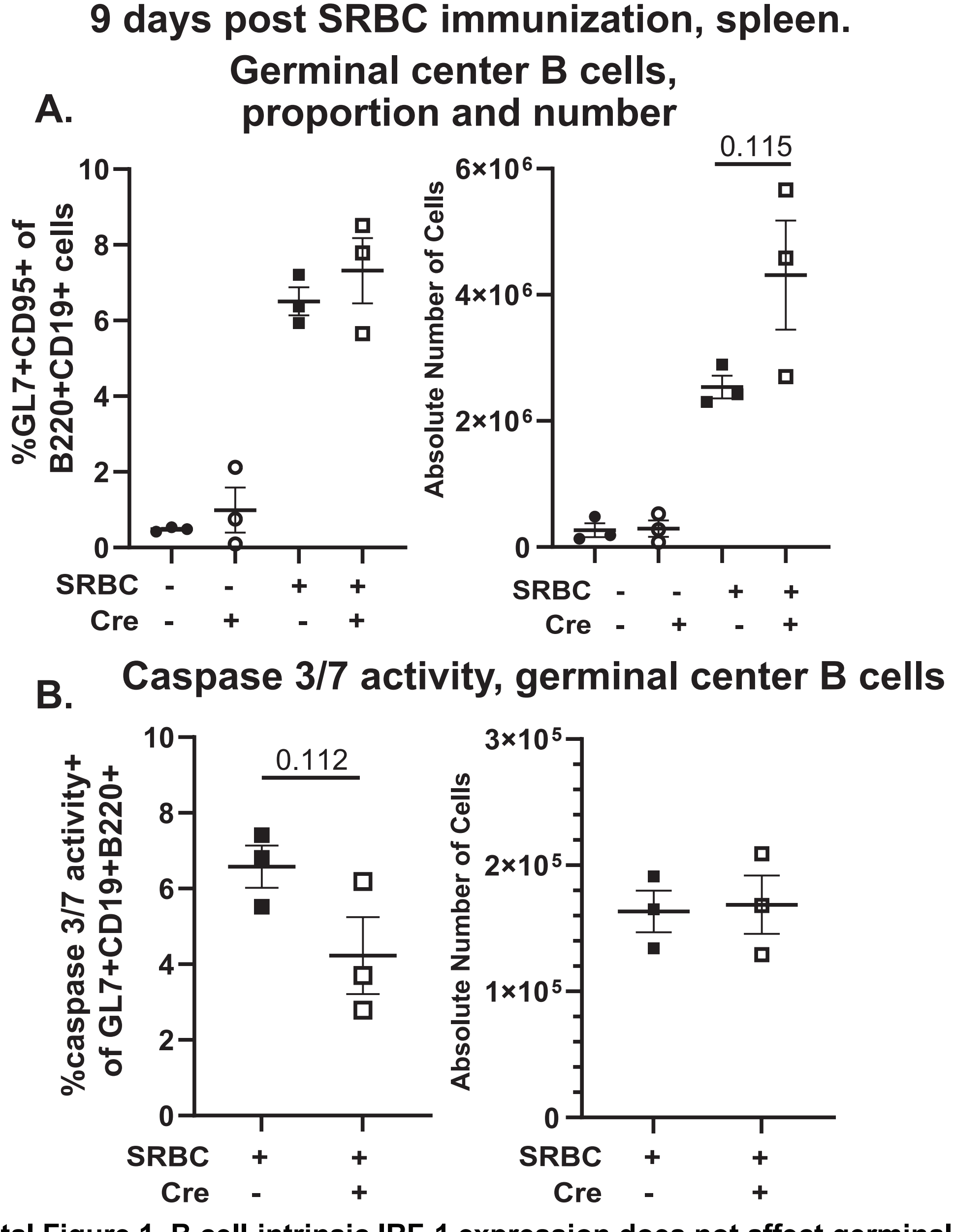

Supplemental Figure 1. B cell-intrinsic IRF-1 expression does not affect germinal center response or apoptosis of germinal center B cells following sheep red blood cell immunization. Cre positive and Cre negative mice with genotypes described in Fig. 1 were intraperitoneally immunized with sheep red blood cells or PBS. Splenocytes were analyzed at 9 days post immunization by flow cytometry to determine the proportion and absolute number of germinal center B cells defined as in Fig. 2A (**A**) and caspase 3/7 activity of germinal center B cells as in Fig. 4 (**B**). Each symbol represents an individual animal; p value is indicated.
