# Supplementary material for: B cell-intrinsic IRF-1 and conserved gammaherpesvirus protein kinase cooperate to promote murine gammaherpesvirus-driven germinal center response and splenic latent reservoir
Source: J Virol. 2025 Nov 20;99(12):e01375-25. doi: 10.1128/jvi.01375-25 (PMC12724244; doi:10.1128/jvi.01375-25)
Supplement: Figure S2 — Gating strategy for DNA damage response marker. [file jvi.01375-25-s0002.pdf]

Supplemental Figure 2

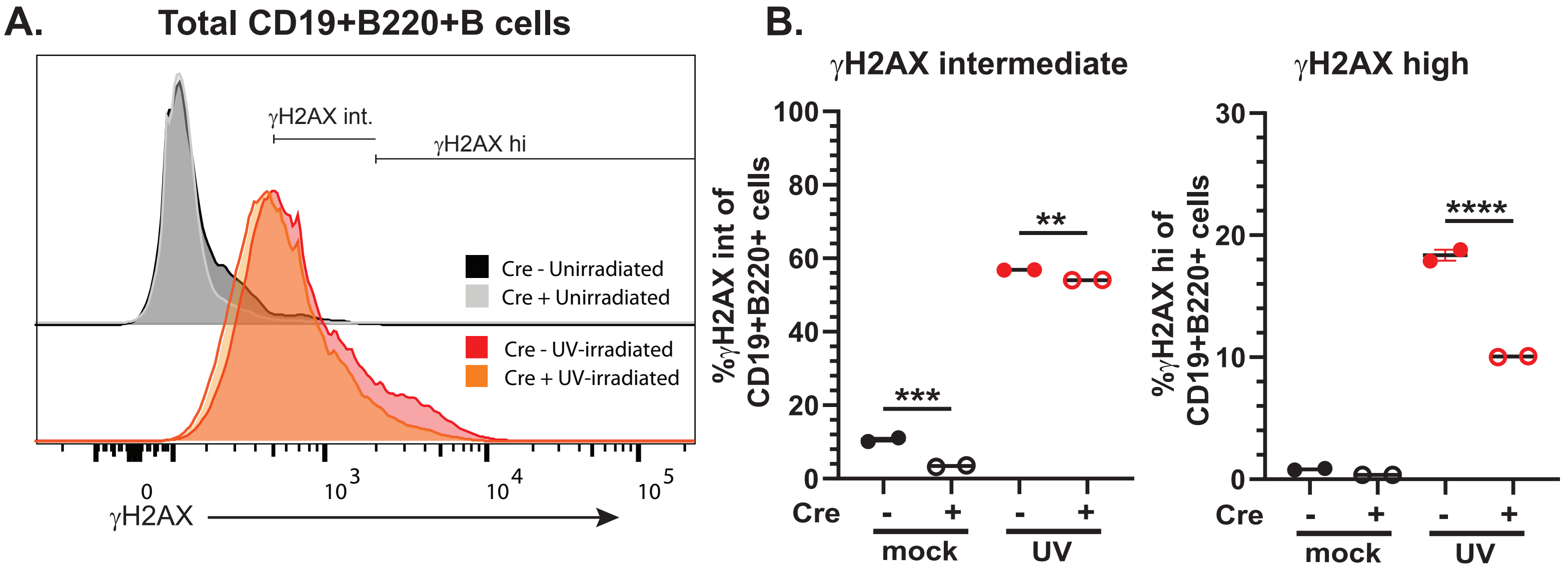

Supplemental Figure 2. γH2AX flow cytometry gating. Splenocytes of naïve Cre negative and Cre positive mice were mock or UV-irradiated and cultured for 3 hours with subsequent analyses of γH2AX levels by flow cytometry. Splenocytes were pre-gated on total B cells (CD19+B220+) and then on γH2AX. **A.** Gating strategy to identify B cells with γH2AX intermediate and γH2AX high levels that was subsequently used for data analyses in Figure 5 of the main text. **B.** Proportion of total splenic B cells (mock- or UV-irradiated), each symbol represents an individual spleen.
